# The membrane-spanning 4-domains, subfamily A (*MS4A*) gene cluster contains a common variant associated with Alzheimer's disease

**DOI:** 10.1186/gm249

**Published:** 2011-05-31

**Authors:** Carmen Antúnez, Mercè Boada, Antonio González-Pérez, Javier Gayán, Reposo Ramírez-Lorca, Juan Marín, Isabel Hernández, Concha Moreno-Rey, Francisco Jesús Morón, Jesús López-Arrieta, Ana Mauleón, Maitée Rosende-Roca, Fuensanta Noguera-Perea, Agustina Legaz-García, Laura Vivancos-Moreau, Juan Velasco, José Miguel Carrasco, Montserrat Alegret, Martirio Antequera-Torres, Salvadora Manzanares, Alejandro Romo, Irene Blanca, Susana Ruiz, Anna Espinosa, Sandra Castaño, Blanca García, Begoña Martínez-Herrada, Georgina Vinyes, Asunción Lafuente, James T Becker, José Jorge Galán, Manuel Serrano-Ríos, Enrique Vázquez, Lluís Tárraga, María Eugenia Sáez, Oscar L López, Luis Miguel Real, Agustín Ruiz

**Affiliations:** 1 Dementia Unit, University Hospital Virgen de la Arrixaca, Ctra. Madrid-Cartagena, Murcia, s/n - 30120 El Palmar, Spain; 2 Alzheimur Foundation, Avda. Juan Carlos, Building Cajamurcia, Murcia, 30100, Spain; 3 Memory Clinic of Fundació ACE, Institut Català de Neurociències Aplicades, Calle del Marqués de Sentmenat, Barcelona, 35-3708029, Spain; 4 Hospital Universitari Vall d'Hebron - Institut de Recerca, Universitat Autònoma de Barcelona (VHIR-UAB), Carretera bellaterra, Barcelona, S/N 08290 Cerdanyola del Vallès, Spain; 5 Department of Structural Genomics, Neocodex, Avda. Charles Darwin, Sevilla, s/n 41092, Spain; 6 Memory Unit, University Hospital La Paz-Cantoblanco, Paseo Castellana, 261, Madrid, 28046, Spain; 7 Alzheimer's Disease Research Center, Departments of Neurology, Psychiatry and Psychology, University of Pittsburgh School of Medicine, 200 Lothrop Street, Pittsburgh PA, PA 15213-2536, USA; 8 Diabetes Research Laboratory, Biomedical Research Foundation, University Hospital Clínico San Carlos, E - 28040, Madrid, Spain

## Abstract

**Background:**

In order to identify novel loci associated with Alzheimer's disease (AD), we conducted a genome-wide association study (GWAS) in the Spanish population.

**Methods:**

We genotyped 1,128 individuals using the Affymetrix Nsp I 250K chip. A sample of 327 sporadic AD patients and 801 controls with unknown cognitive status from the Spanish general population were included in our initial study. To increase the power of the study, we combined our results with those of four other public GWAS datasets by applying identical quality control filters and the same imputation methods, which were then analyzed with a global meta-GWAS. A replication sample with 2,200 sporadic AD patients and 2,301 controls was genotyped to confirm our GWAS findings.

**Results:**

Meta-analysis of our data and independent replication datasets allowed us to confirm a novel genome-wide significant association of AD with the membrane-spanning 4-domains subfamily A (*MS4A*) gene cluster (rs1562990, *P *= 4.40E-11, odds ratio = 0.88, 95% confidence interval 0.85 to 0.91, *n *= 10,181 cases and 14,341 controls).

**Conclusions:**

Our results underscore the importance of international efforts combining GWAS datasets to isolate genetic loci for complex diseases.

## Background

Alzheimer's disease (AD) is the most common neurodegenerative pathology afflicting humans. The prevalence of AD is rapidly growing due to a continuous increase in life expectancy in developed countries [1]. AD is considered a complex neurodegenerative disorder that causes a progressive neuronal loss in the brain, resulting in a devastating cognitive phenotype, which ends with the death of the patient.

Although its etiology is poorly understood, genetic factors seem to play a pivotal role in AD. In fact, three genes containing multiple full penetrance mutations, *APP *(amyloid precursor protein), *PSEN1 *(presenilin 1) and *PSEN2 *(presenilin 2), have been described for Mendelian AD [2-4]. A non-necessary, non-sufficient common allele near the *APOE *(apolipoprotein E) transcript is almost universally associated with non-Mendelian AD [5]. In spite of research efforts in AD genetics, until very recently no other genetic risk factor has been consistently associated with the AD phenotype. However, recent advances in genome wide association study (GWAS) techniques have permitted the isolation of four uncontroversial meta-GWAS-significant (*P *< 5 × E-8) genetic markers associated with AD, which are located near the *CLU *(clusterin), *PICALM *(phosphatidylinositol binding clathrin assembly protein), *CR1 *(complement component (3b/4b) receptor 1) and *BIN1 *(bridging integrator 1) genes [6-8]. No other result derived from genetic studies has been consistently validated for AD other than these loci.

## Materials and methods

### Samples and datasets

In order to identify new AD-associated SNPs, we designed a new case-control GWAS in the Spanish population. We genotyped 1,128 individuals using the Affymetrix Nsp I 250 K chip as previously described [9]. A sample of 327 sporadic AD patients diagnosed as possible or probable AD in accordance with the criteria of the National Institute of Neurological and Communicative Disorders and Stroke and the Alzheimer's Disease and Related Disorders Association (NINCDS-ADRDA) [10] by neurologists at the Virgen de Arrixaca University Hospital in Murcia (Spain) and 801 controls with unknown cognitive status from the Spanish general population were included in our initial study. Mean (standard deviation (SD)) age at recruitment was 79.1 (6.8) years in cases and 52.0 (8.9) in controls. The corresponding number (percentage) of female samples was 228 (71.5%), and 348 (45.4%), respectively. Mean (SD) age at AD diagnosis in cases was 76.2 (6.9) years. Informed consent was obtained from each blood donor. Institutional review board approval for this research was obtained from the regional Ministry of Health (Comunidad Autónoma de Murcia) and conforms to the World Medical Association's Declaration of Helsinki.

To increase the power of our study to detect small genetic effects, we combined our results with those of four other public GWASs, including the Alzheimer's Disease Neuroimaging Initiative (ADNI) longitudinal study, the GenADA study, the National Institute of Aging (NIA) Genetic Consortium for Late Onset Alzheimer's Disease study, and the Translational Genomics Research Institute (TGEN) GWAS [11-14]. The ADNI longitudinal study, which is aimed at identifying biomarkers of AD using the Illumina 610 Quad platform and extensive neuroimaging techniques. A total of 187 early AD cases and 229 elderly controls were initially identified to be included in this study [15]. ADNI data used in the preparation of this article were obtained from the ADNI database [16]. The ADNI was launched in 2003 by the NIA, the National Institute of Biomedical Imaging and Bioengineering (NIBIB), the Food and Drug Administration (FDA), private pharmaceutical companies and non-profit organizations as a $60 million, 5-year public-private partnership. The primary goal of ADNI has been to test whether serial magnetic resonance imaging (MRI), positron emission tomography (PET), and other biological markers are related to the progression of mild cognitive impairment and early AD. Determination of sensitive and specific markers of very early AD progression is intended to aid researchers and clinicians to develop new treatments and monitor their effectiveness, as well as reduce the time and cost of clinical trials. The Principal Investigator of this initiative is Michael W Weiner, MD (VA Medical Center and University of California - San Francisco). ADNI is the result of efforts of many co-investigators from a broad range of academic institutions and private corporations, and subjects have been recruited from over 50 sites across the US and Canada. The initial goal of ADNI was to recruit 800 adults aged 55 to 90 years to participate in the research - approximately 200 cognitively normal older individuals to be followed for 3 years, 400 people with mild cognitive impairment to be followed for 3 years and 200 people with early AD to be followed for 2 years. For up-to-date information, visit ADNI's webpage [16]. The GenADA study includes 801 cases meeting the NINCDS-ADRDA and DSM-IV criteria for probable AD and 776 control subjects without family history of dementia that were genotyped using the Affymetrix 500 K GeneChip Array set [12,17]. The NIA Genetic Consortium for Late Onset Alzheimer's Disease study originally included 1,985 cases and 2,059 controls genotyped with the Illumina Human 610 Quad platform [13]. However, using family trees provided, we excluded all related controls and kept only one case per family. A total of 1,077 cases and 876 unrelated controls were eligible for our study. The TGEN GWAS study included 643 late onset AD cases and 404 controls from a neuropathological cohort and 197 late onset AD cases and 114 controls from a clinical cohort all genotyped with the Affimetrix 500 K GeneChip Array set [11].

Aggregated data from Harold *et al*. [7] and Hu *et al*. [18] were also used as '*in silico*' replication studies. Available data from Harold *et al*. include allelic odds ratio (OR) estimates and *P*-values for the 731 top signals from their study of 3,941 cases and 7,848 controls. A comprehensive list of allelic OR estimates and *P*-values for 451,001 SNPs was obtained from the supplementary material of Hu *et al*. These data correspond to the GWAS described in their manuscript that includes 1,034 cases and 1,186 controls.

Finally a replication sample with 2,200 sporadic AD patients diagnosed as possible or probable AD in accordance with NINCDS-ADRDA criteria by neurologists at Fundació ACE in Barcelona (Spain) and Hospital de Cantoblanco (Madrid), along with 2,301 general population controls was used. Mean (SD) age at recruitment in this sample was 82.0 (7.7) years in cases and 54.7 (12.4) in controls. The corresponding number (percentage) of female samples was 1,559 (71.0%), and 1,540 (67.1%), respectively. Mean (SD) age at AD diagnosis was 77.9 (7.6) years.

### GWAS quality control analyses

We performed extensive quality control on the five datasets with individual genotypes included in the analysis (Murcia, ADNI, GenADA, NIA, TGEN) using Affymetrix Genotyping Console software and Plink [19]. For our genotyped samples, only individuals with a sample call rate above 93% were later re-called with the Bayesian Robust Linear Model with Malalanobis (BRLMM) distance algorithm, ran with default parameters, which improves call rates in most samples. Self-reported sex was compared to sex assigned by chromosome X genotypes, and discrepancies were resolved or samples removed. For all datasets, the program Graphical Representation of Relationships (GRR) [20] was used to check sample relatedness and to correct potential sample mixups, duplications, or contaminations. SNPs were selected to have a call rate above 95% (in each case, control, and combined group, within each dataset), and a minor allele frequency above 1% (again in each case, control, and combined group, within each dataset). SNPs that deviated grossly from Hardy-Weinberg equilibrium (*P*-value < 10-4) in control samples were also removed. We also removed SNPs with a significantly different rate of missingness (*P*-value < 5 × 10-4) between case and control samples within each dataset.

To ensure all SNPs across all datasets were typed according to the same DNA strand, each dataset was normalized using HapMap phase 2 data as the reference set. We merged each study with the HapMap CEU samples and compared genotype calls. SNP calls were flipped (if typed on the opposite strand) or removed (if the strand could not be undoubtedly assigned) as necessary. We also removed SNPs that were significantly associated with 'study status'. That is, we labeled control individuals from each study as cases and HapMap CEU individuals as controls, and removed SNPs with *P*-values < 10-6 in a test for association.

### Principal components analysis

Principal components analysis was carried out with EIGENSOFT [21,22] to evaluate population admixture within each population, and to identify individuals as outliers. We ran the SMARTPCA program with default parameters, excluding chromosome X markers. To minimize the effect of linkage disequilibrium in the analysis, we also excluded markers in high linkage disequilibrium (with the indep-pairwise option in Plink) and long-range linkage disequilibrium regions reported previously or detected in our population. Individuals identified as outliers (six SDs or more along one of the top ten principal components) were removed from all subsequent analyses. Principal component analysis was run within each dataset, and also together with other HapMap European and worldwide populations to detect individuals of different ethnicities.

### Imputation

Since different platforms were used in the five GWASs analyzed, we imputed genotypes using HapMap phase 2 CEU founders (*n *= 60) as a reference panel using two different methodologies: Plink [19] and Mach [23]. Genome-wide imputation was carried out with plink, and genotype calls with high quality scores were used in subsequent association analyses. Best association results were also imputed with Mach 1.0 to confirm the consistency of imputed genotypes.

After all these quality control and preparatory steps, the Murcia study kept 1,034,239 SNPs for 319 cases and 769 controls; the ADNI dataset kept 1,794,894 SNPs for 164 cases and 194 controls; the GenADA dataset kept 1,436,577 SNPs for 782 cases and 773 controls; the NIA dataset kept 1,738,663 SNPs for 987 cases and 802 controls.; and the TGEN dataset contained 1,237,568 SNPs in 757 cases and 468 controls. A total of 696,707 SNPs were common to all GWASs whereas 1,098,485 and 1,951,797 SNPs were common to at least four and two studies, respectively.

### Replication genotyping

The *MS4A *(membrane-spanning 4-domains, subfamily A) cluster polymorphism rs1562990 was genotyped in 2,200 cases and 2,301 controls from the Spanish population using real-time PCR coupled to fluorescence resonance energy transfer (FRET). Primers and probes employed for these genotyping protocols are summarized in Additional file 1. The protocols were performed in the LightCycler^® ^480 System instrument (Roche Diagnostics, Indianapolis, IN, USA). Briefly, PCR reactions were performed in a final volume of 20 μl using 20 ng of genomic DNA, 0.5 μM of each amplification primer, 0.20 μM each detection probe, and 4 μL of LC480 Genotyping Master (5X, Roche Diagnostics). We used an initial denaturation step of 95°C for 5 minutes, followed by 45 cycles of 95°C for 30 s, 55°C for 30 s, and 72°C for 30 s. Melting curves were 95°C for 2 minutes (ramping rate 4.4°C/s), 62°C for 30 s (ramping rate of 1°C/s), 40°C for 30 s (ramping rate of 1°C/s), and 68°C for 0 s (ramping rate of 0.15°C/s). In the last step of each melting curve, a continuous fluorimetric register was performed by the system at one acquisition register per degree celsius. Melting peaks and genotype calls were obtained by using the LightCycler^® ^480 software (Roche). In order to confirm genotypes, selected PCR amplicons were bi-directionally sequenced using standard capillary electrophoresis techniques.

### Association analysis

Unadjusted single-locus allelic (1 degree of freedom) association analysis within each independent sample, and of the combined sample, was carried out using Plink. We combined data from these five GWAS datasets using the meta-analysis tool in Plink selecting only those markers common to at least four of these studies (1,098,485 SNPs). The most promising and consistent results from these single-locus analyses were compared to the aggregated estimates available from Harold *et al*. [7]and Hu *et al*. [18]. Finally, a replication sample of 2,200 cases and 2,301 controls from the Spanish population was used to validate rs1562990. Although the main results of the study are unadjusted estimates and *P*-values from the allelic test, multivariate logistic regression models were also used to adjust estimates for the combined Spanish samples (Murcia GWAS and the replica) by age, sex, and APOE E+ status using the Logistic option in Plink. A final meta-analysis and Forest plot for the marker rs1562990, including the five original GWASs plus the two '*in silico*' replicas and the final replica, was done with the Stata 10.0 (College Station, TX, USA) metan command.

## Results and discussion

**Figure 1:**
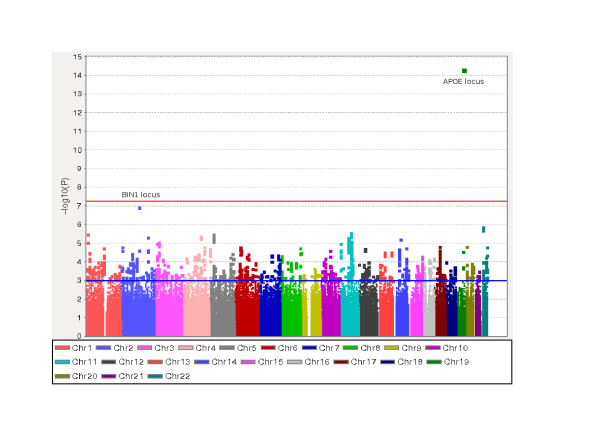
**Manhattan plot of meta-analysis of five GWASs (Murcia, ADNI, GenADA, NIA, and TGEN), including a total of 3,009 cases and 3,006 controls**. A total of 696,707 SNPs were common to all GWASs whereas 1,098,485 SNPs were common to at least four studies.

The meta-analysis of the five GWASs (Murcia, ADNI, GenADA, NIA, and TGEN) included a total of 3,009 cases and 3,006 controls. A total of 696,707 SNPs were common to all GWAS whereas 1,098,485 SNPs were common to at least four. Figure 1 shows a Manhattan plot with the results of this GWAS meta-analysis. We identified several signals, most of them found in previously reported AD loci (Additional file 2). The only GWAS-significant result (*P *= 4.71 × 10-15) corresponded to rs10402271 in chromosome 19, a marker located 78 kb upstream of the *APOE *locus. Other suggestive signals were located in chromosome 2 (rs7561528, located 25 kb downstream of the *BIN1 *locus), chromosome 22 (rs7561528 and rs13447284), and multiple regions within chromosome 11. In fact, among the top 100 markers, 45 were located on chromosome 11 (Additional file 3). Chromosome 11 contains several independent suggestive association signals, including the *HBG2 *(hemoglobin, gamma G) locus (peak association at rs10838245, *P *= 1.04E-5), *MSE4A *gene family cluster (peak association at rs7626344, *P *= 5.48E-6), *GAB2 *(GRB2-associated binding protein 2; rs450128, *P *= 2.79E-6), downstream *PICALM *(rs4944558, *P *= 1.50E-4), and putative downstream gene BC038205 (rs7935502, *P *= 7.47E-5).

We then conducted an '*in silico*' replication of our results using aggregated data from Harold *et al*. [7] (which includes the top 731 signals from their study, many of them also located in chromosome 11) and Hu *et al*. [18] (a comprehensive rank of 451,001 SNPs genotyped in their GWAS). Although limited by the number of SNPs available from these studies, the new meta-analysis yielded quite interesting results, with a total of 17 markers above the GWAS significance level (Additional file 4). Several signals belonged to known AD loci: *APOE *with eight SNPs, *PICALM *(three SNPS, the most significant being rs536841, *P *= 2.96E-9), *CLU *(rs569214, *P *= 4.11E-8), and *BIN1 *(rs744373, *P *= 2.13E-9). Most important, we found four SNPs that belong to a region in chromosome 11q12 not previously reported as GWAS significant for AD. The new peak for AD is located within the *MS4A *cluster and the most significant SNP was rs1562990 (OR 0.87; *P *= 3.01E-10).

Since we have previously published replication studies of *APOE*, *CLU*, *PICALM *and *BIN1 *signals in the Spanish population [8,24], we decided to replicate only rs1562990 in 2,200 cases and 2,301 controls from the this population. Importantly, the result of this new independent replica was fully consistent, yielding a significant OR of 0.90 (95% confidence interval (CI) 0.83 to 0.98; *P *= .01). Detailed results for the original Spanish GWAS dataset, Spanish replica sample, and the combined Spanish dataset are described in Additional file 5. We fitted a multivariate logistic regression model for the combined Spanish sample in which we adjusted for age, sex and *APOE*. The adjusted OR estimate was virtually unchanged (OR 0.87; 95% CI 0.74 to 1.04; *P *= 0.12), suggesting that the observed effect is not influenced by age, sex or *APOE *in our series.

**Figure 2:**
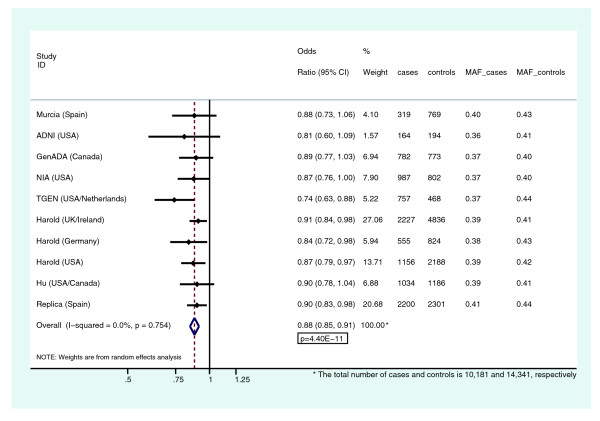
**Meta-analysis and Forest plot of rs1562990, reporting odds ratio (OR) with 95% confidence interval (CI), study-specific weight, sample size and minor allele frequency (MAF) in cases and controls, for each study**. The figure shows the remarkable consistency of the OR across studies.

Finally, combining this new replication in a final meta-analysis together with the five original GWASs and the two '*in silico*' replications yields an OR of 0.88 (95% CI 0.85 to 0.91; *P *= 4.4E-11), which exceeds the accepted threshold for testing multiple comparisons (that is, *P *< 5E-8). A total of 10,181 cases and 14,341 controls are included in this combined analysis. The magnitude of effect is consistent across studies, with all ten estimates between 0.74 and 0.91 (Figure 2).

Our results point to the existence of a new AD locus located within the *MS4A *cluster at 11q12. Coincidentally, during the drafting of this manuscript two independent articles emerged reaching similar conclusions regarding *MS4A *cluster involvement in AD [25,26]. Certainly, the SNP markers described in the three studies are different, but they are only 83,871 bp apart. However, our signal is closer to rs4938933 (reported by Naj *et al*. [27]), which is only 9 kb centromeric to rs1562990. In any case, peak markers observed in these studies are located in the same haplotypic block and have identical effect size and direction, which strongly suggest that they are tracking the same functional variant.

It is important to mention that sample overlapping exists between these studies. Nonetheless, at least three full datasets contained in our study (comprising 7,809 individuals, 31%) do not overlap with previous published works. Importantly, meta-analysis using only these non-overlapping samples also rendered a significant association with the *MS4A *region (OR = 0.897; 95% CI 0.838 to 0.961; *P *= 0.0018). Therefore, our study could be considered an independent replication of the involvement of the *MSA4A *gene cluster in AD. The concurrence of three independent studies reaching the same conclusion by employing different SNP platforms, imputation methods and datasets underscores the strength and consistency of this new AD locus, at least in European populations. Further studies will be necessary to corroborate its involvement in AD etiology in other ethnic groups.

The *MS4A *family includes at least 16 paralogues. Each gene has been probably generated by an ancestral cascade of intrachromosomal duplications during vertebrate evolution. Unfortunately, this gene family is poorly characterized, although a role in immunity has already been shown for several members this cluster, including *MS4A1 *(CD20), *MS4A2 *and *MS4A4B *[28]. However, the function in humans of many other members remains obscure and a more general involvement of *MS4A *family members as ion channel adaptor proteins in non-immune tissues is suspected [28].

The rs1562990 marker maps between *MS4A4E *and *MS4A4A *members of the cluster. However, we detected a critical linkage disequilibrium haplotype block spanning 163 kb that comprises three members of the family (*MS4A2*, *MS4A6A*, and *MS4A4*) and the top four meta-GWAS-significant markers (Additional file 4). With the available data it is difficult to determine the precise location of the functional variant associated with AD, or even which gene could be the best candidate for AD etiology. Furthermore, it may be the case that a functional non-coding variant within the cluster might alter, by *cis*-regulation, the function of other members of the cluster simultaneously. Re-sequencing and functional studies of candidate mutations could help resolve this question.

The most centromeric gene within the critical block, *MS4A2*, encodes a protein that binds to the Fc region of immunoglobulin epsilon. *MS4A2 *seems responsible for initiating the allergic response by binding of allergen to receptor-bound IgE, which leads to cell activation and the release of mediators (such as histamine). This signal cascade is responsible for the manifestations of allergy [29]. Indeed, polymorphisms within the *MS4A2 *gene have been associated with susceptibility to aspirin-intolerant asthma [30], and some epidemiological studies suggest a link between asthma and AD [31]. Consequently, a hypothetical link between *MS4A2 *and AD would add new evidence in favor of the AD neuroinflammatory hypothesis, suggesting a role for the immune system in the pathogenesis of AD. The other genes within the candidate block are poorly characterized and it is not easy to delineate a plausible hypothesis for them yet.

### Data access

GWAS data from Spanish patients is available for qualified researchers after institutional review board approval by the Comunidad Autónoma de la Región de Murcia (Spain). Send requests to Dr Carmen Antúnez Almagro mcarmen.antunez@carm.es.

## Conclusions

We report a new genetic locus associated with AD. Our work underscores the importance of the combination of new GWAS data with existing datasets in order to identify novel signals that can only emerge through meta-analysis. We are confident that the increasing sample size of GWASs, the growing number of publicly available GWAS datasets, the higher marker density and the development of novel strategies for GWAS data analysis will help isolate novel genetic signals related to AD in the future and might contribute to decreasing the missing piece of heritability in neurodegenerative disorders.

## Abbreviations

AD: Alzheimer's disease; ADNI: Alzheimer's Disease Neuroimaging Initiative; bp: base pair; CI: confidence interval; GWAS: genome-wide association study; kb: kilobase; Mb: megabase; MS4A: membrane-spanning 4-domains, subfamily A; NIA: National Institute on Aging; NINCDS-ADRDA: National Institute of Neurological and Communicative Disorders and Stroke and the Alzheimer's Disease and Related Disorders Association; OR: odds ratio; PCR: polymerase chain reaction; SD: standard deviation; SNP: single nucleotide polymorphism; TGEN: Translational Genomics Research Institute.

## Competing interests

RR-L, FJM, JV, JMC, LMR, AG-P, JG, CM-R, ARo, IB, JJG, MES, EV, and AR are employees of Neocodex SL. LMR, EV and AR are shareholders in Neocodex SL. The remaining authors declare that they have no competing interests.

## Authors' contributions

Phenome characterization, database and Biobank construction: CA, MB, JM, IH, CMR, JL-A, AM, MR-R, FN-P, AL-G, LV-M, MA, MA-T, SM, SR, AE, SC, BG, BM-H, GV, AL, JTB, OLL, MS-R, LT, EV, ARo, LMR, AR. Clinical research oversight (Spanish series): CA, MB, IH, JM, OLL, JTB. DNA management and genome analysis: RR-L, FJM, JV, JMC, JJG, MES, LMR, AR. Bioinformatics, statistical analysis and IT support: AG-P, JG, RRL, CM-R, ARo, IB, JJG, MES, AR. Writers: AG-P, JG, JTB and AR with contributions from all authors. Project design and funding: CA, MB, LT, EV, LMR, AR. Project oversight: CA, MB, AR. All authors read and approved the final manuscript.

## Supplementary Material

Additional file 1 **Table S1 - primers and probes employed for Real-time detection of MS4A cluster rs1562990 marker**. Molecular Information for rs1562990 genotyping. Click here for file

Additional file 2 **Table S2 - top 100 results in the meta-analysis including five initial GWAS**. Best results obtained in our study. CHR, chromosome; A1, allele 1; A2, allele 2; N, number of studies in the meta-analysis contributing to the overall estimate of the marker; P, *P*-value from fixed effects model; P(R), *P*-value from random effects model; OR, pooled odds ratio estimate from fixed effects model; OR(R), pooled odds ratio estimate from random effects model; Q, *P*-value for Cochrane's Q statistic; I, I^2 ^heterogeneity index. Click here for file

Additional file 3 **Table S3 - GWAS plus aggregated data from Harold *et al*. and Hu *et al***. GWAS-significant markers obtained after *in silico *replications. CHR, chromosome; A1, allele 1; A2, allele 2; N, number of studies in the meta-analysis contributing to the overall estimate of the marker; P, *P*-value from fixed effects model; P(Random), *P*-value from random effects model; OR, pooled odds ratio estimate from fixed effects model; OR(Random), pooled odds ratio estimate from random effects model; Q, *P*-value for Cochrane's Q statistic; I, I^2 ^heterogeneity index. Click here for file

Additional file 4 **Table S4 - *MS4A *rs1562990 minor allele frequency (MAF), Genotype distribution, effect estimates, and significance in the Spanish series**. Table describing the results of *MS4A *cluster region in the Spanish population. Click here for file

Additional file 5 **Figure S1 - Manhattan plot with results from the meta-analysis of the five initial GWASs for markers in chromosome 11**. MetaGWAS results obtained for chromosome 11. Click here for file

Additional file 6 **File S1 - Alzheimer's Disease Neuroimaging initiative (ADNI) active investigators**. Full list of ADNI co-investigators (alphabetical order). Click here for file
