# Supplementary material for: The membrane-spanning 4-domains, subfamily A (MS4A) gene cluster contains a common variant associated with Alzheimer's disease
Source: Genome Med. 2011 May 31;3(5):33. doi: 10.1186/gm249 (PMC3219074; doi:10.1186/gm249)
Supplement: Additional file 1 — Table S1 - primers and probes employed for Real-time detection of MS4A cluster rs1562990 marker. Molecular Information for rs1562990 genotyping. [file gm249-S1.DOC]

**Table S1.- Primers and probes employed for Real-time detection of MS4A cluster rs1562990 marker**

| PCR Primers | Forward | GGAGCTCAGTCAAGCATTCC |
| --- | --- | --- |
|  | Reverse | ACCAATGTGCTCCACTCCAG |
| FRET Probes | Sensor | [Cy5]CCTGAACGATCAAAGGCT[Phos] |
|  | Anchor | GGATGAAGCACCACACACAAG[Flc] |

Table legends: [Cy5], Fluorochrome Cy5; [Phos], Phosphorothioate; [Flc] , Fluorescein. All sequences are given in 5’ to 3’ direction.
