# Supplementary material for: The membrane-spanning 4-domains, subfamily A (MS4A) gene cluster contains a common variant associated with Alzheimer's disease
Source: Genome Med. 2011 May 31;3(5):33. doi: 10.1186/gm249 (PMC3219074; doi:10.1186/gm249)
Supplement: Additional file 5 — Figure S1 - Manhattan plot with results from the meta-analysis of the five initial GWASs for markers in chromosome 11. MetaGWAS results obtained for chromosome 11. [file gm249-S5.DOC]

**Table S4. MS4A rs1562990 MAF, Genotype distribution, effect estimates, and significance in the Spanish series.**

|  | MAF cases | MAF controls | Genotypes cases | Genotyopes controls | OR (95%CI) | p-value |
| --- | --- | --- | --- | --- | --- | --- |
| GWAS Murcia | 0,40 | 0,43 | 110/164/45 | 249/378/142 | 0.88 (0.73-1.06) | 0.164 |
| Replica | 0,41 | 0,44 | 781/1020/399 | 695/1190/416 | 0.90 (0.83-0.98) | 0.012 |
| Combined | 0,41 | 0,44 | 891/1184/444 | 944/1568/558 | 0.90 (0.83-0.97) | 0.006 |

Table legends: MAF: Minor allele frequency; OR:Pooled Odds Ratio estimate from fixed effects model; CI: Confidence Interval.
