# Supplementary material for: The membrane-spanning 4-domains, subfamily A (MS4A) gene cluster contains a common variant associated with Alzheimer's disease
Source: Genome Med. 2011 May 31;3(5):33. doi: 10.1186/gm249 (PMC3219074; doi:10.1186/gm249)
Supplement: Additional file 6 — File S1 - Alzheimer's Disease Neuroimaging initiative (ADNI) active investigators. Full list of ADNI co-investigators (alphabetical order). [file gm249-S6.DOC]

**File S1: Alzheimer’s Disease Neuroimaging initiative (ADNI) active investigators.**

Saradha A, Abdi H, Abdulkadir A, Abeliovich A, Abellan van Kan G, Abner E, Acharya D, Agrusti A, Agyemang A, Ahdidan J, Ahmed S, Ahn JE, Aisen P, Aksu Y, Al-Akhras M, Alarcon M, Alberca R, Alexander G, Alexander D, Alin A, Almeida F, Amlien I, Anand S, Anderson D, Andrew M, Angersbach S, Anjum A, Aoyama E, Arfanakis K, Armor T, Arnold S, Arunagiri V, Asatryan A, Ashe-McNalley C, Ashiga H, Assareh A, Le Page A, Avants B, Avinash G, Aviv R, Awasthi S, Ayan-Oshodi M, Babic T, Baek Y, Bagci U, Bai S, Baird G, Baker J, Banks S, Bard J, Barnes J, Bartlett J, Bartzokis G, Barua N, Bauer C, Bayley P, Beck I, Becker J, Becker JA, Beckett L, Bednar M, Beg MF, Bek S, Belaroussi B, Belmokhtar N, Bernard C, Bertram L, Bhaskar U, Biffi A, Bigler E, Bilgic B, Bishop C, Bishop C, Bittner D, Black R, Bogorodzki P, Bokde A, Bonner-Jackson A, Boppana M, Bourgeat P, Bowes M, Bowman D, Bowman G, Braskie M, Braunewell K, Breitner J, Bresell A, Brewer J, Brickman A, Britschgi M, Broadbent S, Brogren J, Brooks D, Browndyke J, Brunton S, Buchert R, Buchsbaum M, Buckley C, Buerger K, Burger C, Burnham S, Burns J, Burton D, Butman J, Cabeza R, Cairns N, Callhoff J, Callhoff J, Calvini P, Cantillon M, Capella H, Carbotti A, Cardona-Sanclemente LE, Carle A, Carmasin J, Carranza-Ath F, Casabianca J, Casanova R, Cash D, Cedarbaum J, Cella M, Celsis P, Chanu P, Chao L, Charil A, Chemali Z, Chen R, Chen J, Chen G, Chen W, Chen K, Chen S, Chen M, Cheng WC, Cherkas Y, Chertkow H, Cheung C, Cheung V, Chiang G, Chiba K, Chin S, Chisholm J, Cho Y, Choe J, Choubey S, Chowbina S, Christensen AL, Clark D, Clark C, Clarkson M, Clayton D, Clunie D, Coen M, Coimbra A, Coimbra A, Compton D, Coppola G, Coulin S, Cover KS, Crane P, Crans G, Croop R, Crowther D, Crum W, Cui Y, Curry C, Curtis S, Cutter G, Daiello L, Dake M, Dale A, Daliri MR, Damato VD, Darby E, Darkner S, Darkner S, Davatzikos C, Dave J, David R, DavidPrakash B, Davidson J, de Bruijne M, De Meyer G, De Nunzio G, DeCarli C, Dechairo B, DeDuck K, Dehghan H, Dejkam A, Delfino M, Della Rosa PA, Dellavedova L, Delpassand E, Delrieu J, DeOrchis V, Depy Carron D, deToledo-Morrell L, Devanand D, Devanarayan V, Devous M, Diaz-Arrastia R, Bradford D, Ding X, Dinov I, Dobson H, Dodge H, Donohue M, Dore V, Dorflinger E, Dowling M, Dowling M, Duan X, Dubal D, Duchesne S, Duff K, Dukart J, Durazzo T, Dykstra K, Earl N, Edula G, Ekin A, Elcoroaristizabal X, Emahazion T, Emahazion T, Engelman C, Epstein N, Erten-Lyons D, Eskildsen S, Falcone G, Fan L, Fan Y, Farahibozorg S, Farb N, Farnum M, Farrer L, Farzan A, Faux N, Feldman B, Feldman H, Feldman S, Fennema-Notestine C, Fernandes M, Fernandez E, Ferrarini L, Ferreira MJ, Ferrer E, Figurski M, Filipovych R, Fillit H, Finch S, Finlay D, Fiot JB, Flenniken D, Fletcher PT, Fletcher E, Flynn Longmire C, Focke N, Forman M, Forsythe A, Fox S, Fox-Bosetti S, Francis AL, Franco-Villalobos C, Franko E, Freeman S, Friedrich CM, Friesenhahn M, Frings L, Frisoni G, Fritzsche K, Fujimoto Y, Fujiwara K, Fullerton T, Furney S, Gallins P, Galvin B, Gamst A, Gan K, Garcia MT, Garg G, Gaser C, Gastineau E, Gauthier S, Gavett B, Gavidia G, Gazdzinski S, Ge Q, Ge T, Gemme G, Geraci J, Ghassabi Z, Gieschke R, Gil JE, Gill R, Gitelman D, Gleason C, Glymour MM, Godbey M, Goghari V, Gold M, Goldberg T, Goldman J, Gomeni R, Gong S, Gonzales C, Goodro R, Gordon B, Gore C, Gorriz JM, Grachev I, Grandey E, Grasela T, Gratt J, Gray K, Greenberg B, Gregg K, Gregory E, Greicius M, Greve D, Grill J, Gross A, Gross A, Guignot I, Guo J, Guo Q, Guo H, Guo L, Habeck C, Hai Y, Haight T, Hammarstrom P, Hampel H, Han D, Han J, Han T, Hanif M, Hanna Y, Hardy P, Harvey D, Hasan MK, Hayashi T, Hazart A, He H, He Y, Head D, Head D, Heckemann R, Heidebrink J, Henderson D, Henrard S, Herholz K, Hernandez M, Herskovits AZ, Hess C, Hildenbrand M, Hobart J, Hoffman J, Holder D, Hollingworth P, Holmes R, Honigberg L, Hoppin J, Hou Y, Hsu A, Hsu WW, Hu X, Hu Z, Hu W, Huang J, Huang CC, Huang C, Huang S, Huang Y, Huang F, Huang CJ, Huang SP, Hubbard R, Huentelman M, Hui S, Huppertz HJ, Hurko O, Hurt S, Huyck S, Hwang S, Hyun J, Ifeachor E, Iglesias M, Ikari Y, Ikonomidou V, Imani F, Immermann F, Inlow M, Inoue L, Insel P, Irizarry M, Irungu B, Ishibashi T, Ishii K, Ismail S, Ismail S, Ito K, Iturria-Medina Y, Iwatsubo T, Jacobson M, Jacqmin P, Jafari A, Jafari-Khouzani K, Jaffe C, Jagust W, Jagust W, Jara H, Jasperse B, Jedynak B, Jefferson A, Jennings JR, Jessen W, Jia F, Jiang T, Jing H, Johnson J, Johnson S, Johnson DK, Jones R, Juengling F, Juh R, Julin P, Bhaskar U, Kadish B, Kahle-Wrobleski K, Kallam HR, Kamboh MI, Kaneko T, Kaneta T, Kang JH, Karageorgiou E, Karantzoulis S, Karlawish J, Katz E, Kaushik SS, Kauwe J, Kauwe J, Kawakami H, Kazimipoor B, Kelleher T, Kennedy R, Kerchner G, Kerrouche N, Khalil I, Khalil A, Killeen N, Killiany R, Kim JH, Kim H, Kim A, Kim Y, Kim H, Kim S, Kim H, Kim A, Kimberg D, Kimura T, King R, Kirby J, Kirsch W, Klimas M, Kline R, Kling M, Klopfenstein E, Koikkalainen J, Kokomoor A, Kolasny A, Koppel J, Korolev I, Kotran N, Kouassi A, Kowalczyk A, Kozma L, Krams M, Kratzer M, Kuceyeski A, Kuhn FP, Kumar S, Kuo HT, Kuo J, Kurosawa K, Kwon OH, Labrish C, Laforet G, Lai S, Lakatos A, Lam OK, Lampron A, Landau S, Landen J, Lane R, Langbaum J, Langford D, Lanius V, Laxamana J, Le T, Leahy R, Lee JM, Lee V, Lee JH, Lee G, Lee D, Lee N, Lefkimmiatis S, Lemaitre H, Lenfant P, Lenz R, Leoutsakos JM, Lester G, Levey A, Li SJ, Li S, Li W, Li CS, Li X, Li R, Li M, Li L, Li J, Li Y, Li Q, Li G, Liang K, Liang P, Liang L, Liao YL, Lin LC, Lin L, Lin M, Lin AL, Liu S, Liu Y, Liu T, Liu M, Liu X, Liu L, Liu H, Liu P, Liu T, Liu S, Liu D, Lo R, Lobanov V, Loewenstein D, Logovinsky V, Long X, Long Z, Looi J, Lu PH, Lukic A, Lull JJ, Luo X, Lynch J, Ma L, Mackin S, Mada M, Magda S, Maglio S, Maikusa N, Mak HK, Malave V, Maldjian J, Mandal P, Mangin JF, Manjon J, Mantri N, Manzour A, Marambaud P, Marchewka A, Marek K, Markind S, Marshall G, Martinez Torteya A, Mather M, Mathis C, Matoug S, Matsuo Y, Mattei P, Matthews D, McArdle J, McCarroll S, McEvoy L, McGeown W, McGonigle J, McIntyre J, McLaren D, McQuail J, Meadowcroft M, Meda S, Mehta N, Melie-Garcia L, Melrose R, Mendonca B, Menendez M, Meredith J, Merrill D, Mesulam MM, Metti A, Meyer C, Mez J, Mickael G, Miftahof R, Mikhno A, Miller D, Millikin C, Min Y, Mirza M, Mistridis P, Mitchell M, Mitsis E, Mohan A, Moore D, Moradi Birgani P, Moratal D, Morimoto B, Mormino E, Mortamet B, Moscato P, Mueller K, Mueller S, Mueller N, Mukherjee S, Mulder E, Murayama S, Murphy M, Murray B, Musiek E, Myers A, Najafi S, Nazarparvar B, Nazeri A, Nettiksimmons J, Neu S, Ng YB, Nguyen N, Nguyen Xuan T, Nichols T, Nicodemus K, Niecko T, Nielsen C, Notomi K, Nutakki GC, O'Bryant S, O'Neil A, Obisesan T, Oh DH, Oh J, Okonkwo O, Olde Rikkert M, Olmos S, Ortner M, Ostrowitzki S, Oswald A, Ott B, Ourselin S, Ouyang G, Paiva R, Pan Z, Pande Y, Pardo J, Pardoe H, Park H, Park L, Park MH, Park S, Park KH, Park S, Parsey R, Parveen R, Paskavitz J, Patel Y, Patil M, Pawlak M, Payoux P, Pearson J, Peavy G, Pell G, Peng Y, Pennec X, Pepin J, Perea R, Perneczky R, Petitti D, Petrella J, Peyrat JM, Pezoa J, Pham CT, Phillips J, Phillips N, Pierson R, Piovezan M, Podhorski A, Pollari M, Pontecorvo M, Poppenk J, Posner H, Potkin S, Potter G, Potter E, Poulin S, Prasad G, Prenger K, Prince J, Priya A, Puchakayala SR, Puchakayala SR, Qiu R, Qiu A, Qiu W, Qualls CD, Rabie H, Rajeesh R, Rallabandi VP, Ramage A, Randolph C, Rao A, Rao D, Raubertas R, Ray D, Razak H, Redolfi A, Reed B, Reid A, Reilhac A, Reinsberger C, Restrepo L, Retico A, Richards J, Riddle W, Ries M, Rincon M, Rischall M, Rizk-Jackson A, Robieson W, Rocha-Rego V, Rogalski E, Rogers E, Rojas I, Rojas Balderrama J, Romero K, Rorden C, Rosand J, Rosen A, Rosen O, Rosenberg P, Ross D, Roubini E, Rousseau F, Rowe C, Rubin D, Rubright J, Ruiz A, Rusinek H, Ryan L, Saad A, Sabbagh M, Sabuncu M, Sachs M, Sadeghi A, Said Y, Saint-Aubert L, Sakata M, Salat D, Salmon D, Salter H, Samwald M, Sanchez L, Sanders E, Sanders E, Sanjo N, Sarnel H, Sato H, Sato S, Saumier D, Savio A, Sawada I, Saykin A, Schaffer JD, Scharre D, Schegerin M, Schlosser G, Schmand B, Schmansky N, Schmidt M, Schmidt-Wilcke T, Schneider L, Schramm H, Schuerch M, Schwartz E, Schwartz C, Schwarz A, Seethamraju R, Seixas F, Selnes P, Senjem M, Senlin W, Seo SW, Sethuraman G, Sevigny J, Sevigny J, Sfikas G, Sghedoni R, Shah SK, Shahbaba B, Shams S, Shattuck D, Shaw L, Shaw L, Sheela J, Shen W, Shen Q, Shera D, Sherman J, Sherva R, Shi F, Shukla V, Shuler C, Shulman J, Siegel R, Siemers E, Silveira M, Silver M, Silverman D, Sim I, Simmons A, Simmons A, Simoes R, Simon M, Simpson I, Singh SP, Singh N, Siuciak J, Sjogren N, Skinner J, Skup M, Small G, Smith M, Smith B, Smith C, Smyth T, Snow S, Soares H, Soldea O, Solomon P, Solomon A, Som S, Song C, Song M, Sosova I, Soudah E, Soydemir M, Spampinato MV, Spenger C, Sperling R, Spiegel R, Spies L, Squarcia S, Squire L, Staff R, Stern Y, Straw J, Stricker N, Strittmatter S, Stühler E, Styren S, Subramanian V, Sugishita M, Sukkar R, Sun J, Sun Y, Sun Y, Sun J, Sundell K, Suri M, Suzuki A, Svetnik V, Swan M, Takahasi T, Takeuchi T, Tanaka S, Tanchi C, Tancredi D, Tao W, Tao D, Taylor-Reinwald L, Teng E, Terlizzi R, Thames A, Thiele F, Thomas B, Thomas R, Thompson P, Thompson W, Thornton-Wells T, Thorvaldsson V, Thurfjell L, Titeux L, Tokuda T, Toledo JB, Tolli T, Toma A, Tomita N, Toro R, Torrealdea P, Tosun D, Tousian M, Toussaint P, Toyoshiba H, Tractenberg RE, Trittschuh E, Trojanowski J, Truran D, Tsechpenakis G, Tucker-Drob E, Tufail A, Tung J, Turken A, Ueda Y, Ullrich L, Umadevi Venkataraju K, Umar N, Uzunbas G, Van de Nes J, van der Brug M, Van Horn J, Van Leemput K, Van Train K, Van Zeeland A, Vasanawala M, Vemuri P, Verwaerde P, Videbaek C, Vidoni E, Villanueva-Meyer J, Visser PJ, Vitolo O, Vounou M, Wade S, Walhovd KB, Wan H, Wang H, Wang YM, Wang Y, Wang A, Wang L, Wang Y, Wang X, Wang Z, Wang Y, Wang T, Wang A, Wang H, Wang LS, Wang W, Wang L, Ward M, Warfield S, Waring S, Watanabe T, Webb D, Wei L, Weiner M, Wen SH, Wenjing L, Wenzel F, Westlye LT, Whitcher B, Whitlow C, Whitwell J, Wilhelmsen K, Williams D, Wilmot B, Wimsatt M, Wingo T, Wiste H, Wolfson T, Wolke I, Wolz R, Woo J, Woo E, Woods L, Worth A, Worth E, Wouters H, Wu T, Wu YG, Wu L, Wu X, Wyman B, Wyss-Coray T, Xiao G, Xiao L, Xie S, Xu S, Xu Y, Xu YZ, Xu G, Xu J, Yamane T, Yamashita F, Yan Y, Yan Y, Yan P, Yang E, Yang J, Yang QX, Yang Z, Yang G, Yang Z, Yang W, Ye L, Ye BS, Ye J, Ye J, Yee L, Yesavage J, Ying S, Yoo B, Young J, Yu S, Yu D, Yuan G, Yuan K, Yushkevich P, Zaborszky L, Zagorodnov V, Zagorski M, Zawadzki R, Zeitzer J, Zelinski E, Zhang K, Zhang H, Zhang T, Zhang H, Zhang X, Zhang P, Zhang B, Zhang J, Zhang L, Zhang L, Zhang Z, Zhao Q, Zhao J, Zhao P, Zhen X, Zhen X, Zheng Y, Zhijun Y, Zhou B, Zhou S, Zhu W, Zhu H, Zhu W, Zilka S, Zito G, Zou H.
